# Effectiveness of edutainment use in video-based learning on oral health education for school-age children: a randomized clinical trial

**DOI:** 10.1186/s12903-025-05717-9

**Published:** 2025-03-13

**Authors:** Wanwisa Lekaram, Pattarawadee Leelataweewud, Pornpailin Kasemkhun

**Affiliations:** Department of Pediatric Dentistry, Faculty of Dentistry, Mahidol University, No.6, Yothi Road, Ratchathewi District, Bangkok, 10400 Thailand

**Keywords:** Classroom learning, Edutainment, Education, Oral health, School-age children, Video-based learning

## Abstract

**Background:**

Entertainment platforms have become more popular among children since the COVID-19 outbreak. The entertainment designed for education; “edutainment” could be a promising learning tool on oral health education (OHE). This study aimed to evaluate the effectiveness of edutainment use for OHE in school-age children.

**Methods:**

A total of 210 students (age range 9.2–10.8 years) were included. The three-arm parallel randomized controlled trial was conducted in three schools, receiving the same contents of OHE with different learning methods; classroom learning (CL), edutainment in video-based learning with and without repetition at a three-month follow-up (EVBL and EVBL-R). The knowledge, behavior, behavioral intention score, and oral health (OH) status; visble plaue (VPI) and gingival index (GI) were evaluated at baseline, immediate post-intervention, 3, and 6 months. Differences within and between groups for knowledge scores and OH status were analyzed by repeated measures and one-way ANOVA, respectively, and for both behavior and behavioral intention scores, Friedman’s two-way analysis of variance and Kruskal-Wallis test were analyzed, respectively, at a significant level of 0.05.

**Results:**

The knowledge score was significantly higher in CL for two out of three content domains (*p =* 0.01, *p* < 0.001) yet immediately improved within all groups (*p* < 0.001) with the range of 26.58–53.35% vs. 4.12–29.77% of both EVBLs. No significant difference was found in the behavior and behavioral intention scores among groups. EVBL and EVBL-R had significantly improved behavior scores throughout their follow-ups (*p* = 0.017, *p* = 0.006) with the range of 1.19–28.13% vs. 1.90-15.16% of CL and had a significant improvement for VPI (*p* < 0.001) or 32.5-57.08% vs. 36.45–38.79% of CL. There was no significant difference in GI, but it significantly improved only within the EVBL-R group after the repetition.

**Conclusion:**

EVBL was comparable to the CL in encouraging positive behaviors, while the CL was preferable for providing core knowledge. EVBL was more applicable to how-to content, and the repetition at least every three months might be able to promote a better OH status.

**Trial registration:**

The trial was registered in the Thai Clinical Trials Registry under the number TCTR20240816001 on 16/08/2024 (retrospective registration).

**Supplementary Information:**

The online version contains supplementary material available at 10.1186/s12903-025-05717-9.

## Background

Dental caries and gingivitis are the most common oral diseases that affect the appearance, quality of life. Although, dental caries and gingivitis are preventable diseases, parents, who seem to be the most influential on children’s oral health (OH), themselves have restricted knowledge [1]. Thus, school-based oral health education (OHE) was established with the main objective of overcoming the barriers that children and families have faced in accessing dental services and to remove inequalities in OHE between children in different communities [2–5].

School is the appropriate place to promote the OHE, and school-age children are the most effective stage for reinforcing beliefs, attitudes, and skills. Normally, OHE should be directly provided by dentists and other oral health care providers, but due to a shortage of those personnel, school-based OHE still requires assistance from teachers. It was found that school-based OHE with well-trained teachers could provide a long-term benefit, though it added an extra workload for teachers [6].

The most widely used traditional learning method for school-based OHE is cooperative learning, or a learner-centered approach in a classroom environment [7]. Game-based learning has also been applied and it was found to be effective in enhancing OH knowledge and skills in school-age children through adolescents [8–10]. Video-based learning (VBL) was introduced using its simple study design, which required no additional school personnel and was also suitable for a school timetable. According to the studies, it can significantly improve children’s OH knowledge as well as their plaque index [11, 12]. However, it limits the interaction, which makes it difficult to access the understanding of the learner, rely on the technology, and also the production cost that impedes the wide use of VBL in OHE.

Nowadays, the learning behavior of school-age children has changed. They tend to use social media to seek both knowledge and entertainment in order to answer all their questions and entertain themselves at once, especially since the COVID-19 outbreak. Therefore, it could be possible that the combination of education and entertainment, or “edutainment” video, is appropriate to be a promising learning tool in OHE to overcome the limitations of OH personnel shortages and reduce the extra tasks of teachers without bias against the individual capabilities of the knowledge provider, including enhancing the learning ability of children since edutainment can attract the learners through amusement and enjoyment as it has been proven to be effective in the learning process in both children and adults [13, 14]. However, there was no attempt to apply an age appropiate edutainment video on OHE before. Thus, this randomized controlled study aimed to evaluate the effectiveness of edutainment use for OHE in school-age children.

## Methods

### Ethical considerations

Ethical approval was obtained by the Ethical Institutional Review Board (IRB), Faculty of Dentistry and Faculty of Pharmacy, Mahidol University (MU-DT/PY-IRB 2023/DT040) and retrospectively registered in the Thai Clinical Trials Registry (No.TCTR20240816001) on 16/08/2024. Written informed consent was obtained from the parents whose children meet the criteria, and an assent was given by the children. All questions or suspicions were answered.

### Study design and setting

This was a three-arm parallel randomized controlled trial that evaluated the effectiveness of edutainment use for OHE in school-age children. The study was conducted in Nakhon Pathom province, Thailand, during August 2023 to March 2024. The settings were elementary schools located in the main districts.

### Participants and randomization

The sample size calculation was based on the study of Ramezaninia et al. [15] which compared the plaque index in school-age children using different methods of OHE, using G*power software (version 3.1.9.4) with a power and an alpha error set at 0.8 and 0.05, respectively. A sample size of 59 students was required, anticipating a 20% dropout rate; the total sample size in this study was 70 students per group. The inclusion criteria were healthy students who attended in grades 4–5, have never been involved in a similar OHE program in the last 6 months, or receive any extra OHE other than in the national curriculum. Participants had the ability to use a computer, smartphone, or tablet. Special needs students who had learning problems or disabilities reported by the homeroom teacher, participants who had a visible plaquer index (VPI) [16] and a gingival index (GI) [17] equal to zero were excluded. Only medium-sized public elementary schools that had a sound lab were included.

Three medium-sized public elementary schools located in the main districts were included by purposive sampling according to their similar characteristics mentioned previously. The allocation was performed by the external dentist who was not involved in any procedures of the study, the simple randomization was performed using drawing lots to assign only one group per school, as follows: (1) classroom learning (CL); (2) edutainment in video-based learning (EVBL); and (3) EVBL with repetition (EVBL-R). Then the students in each school were recruited by simple random sampling using drawing lots by the same dentist for choosing only one whole class (2–3 classes of each grade level) from grades 4 and 5, respectively.

### Interventions

Knowledge, behavior, and behavioral intention scores from the purposefully developed test questions for this study using multiple-choice and yes-no questions, including OH status (VPI and GI), were the primary outcomes of the trial. The content of the Thai-language test questions was based on elementary school lessons directed by the Bureau of Dental Health, Ministry of Health, Thailand. There were three content domains, which consisted of: general knowledge about dental and gingival diseases, including the necessity of regular dental check-up visits (D-I); oral health care (D-II); and diet (D-III). The content validity, using the index of item objective congruence equal to one, was evaluated by specialists in OH prevention and pediatric dentistry. The test questions were piloted by 21 children of same age range with our participant or 10% of the calculated sample size to be assured that they were all age appropriate. The questions that confused the reader were revised and improved before use. Then, OH status using the VPI and GI was examined by two blinded trained and calibrated dentists as examiners using an examination mattress with a blunt explorer and flat mouth mirror under a headlamp light at the schools.

EVBL was developed based on the content domains of the test questions, using theories of health behavior [18, 19]; the Health Belief Model, Reasoned Action and Planned Behavior, Social Cognitive Theory, and Protection Motivation Theory, which vary in each clip. It was designed to be vocally delivered in the rapping style of hip-hop music that was newly composed with lyrics presented to sing along. A short clip, lasting only one to two minutes. The quality of the production clip and the contents were validated by two experienced pediatric dentists, confirming that it conformed with the core domain lessons from the test questions. Then it was piloted by the same group of children in the test question piloting step. The clip that significantly confused the children was revised and repeated with all the previous steps. The video clips were played by computers and headphones individually at the school sound lab. The video clip links were generated as QR code pictures and were displayed in their classrooms for the purpose of on-demand replaying. The CL lesson was developed from the same contents as the edutainment video clips, delivered as a presentation in a classroom by a full-time teacher without entertaining, yet being able to answer the questions from students if needed. This technique was also evaluated for the quality of teaching materials and methods and validated by the same two experienced pediatric dentists. Both interventions were provided by the same investigator.

At baseline, all participants completed the demographic data and pre-test questions, and were examined for their OH status. After that, the OHE session was provided a week after the baseline data collection. The OHE session was performed once a week per one content domain related to the test questions, with the same objective and content in two different learning methods; CL and EVBL. The toothbrush was given to all groups when the oral health care (D-II) was provided to standardize the toothbrushing. All video clips were allowed to be rerun by the students themselves through the given online entertainment platform. Then, the test questions were repeated at immediate post-intervention (for knowledge and behavioral intention), then at 3-month and 6-month follow-ups, as with the VPI and GI. The repetition using the same clips in the EVBL-R group was provided once at three-month intervals between 3 and 6 months of follow-up, imitating the interval of routine dental recall for high caries risk patients, to evaluate if repetition could enhance outcomes.

### Statistical analysis

Demographic data was evaluated using descriptive statistics and the differences between groups were evaluated by Chi-square test. Differences in OH knowledge scores and status within and between groups were analyzed by repeated measures ANOVA and one-way ANOVA respectively, as they were normally distributed variables. While differences in OH behavior and intention scores within and between groups were analyzed by related-samples Friedman’s two-way analysis of variance and independent-samples Kruskal-Wallis test, respectively. Differences of mean scores among variables within groups were analyzed by independent samples t-Test. The intra- and inter-examiner reliability for OH status examination was calculated by Cohen’s kappa statistics. The level of significance was set as 0.05 for all statistical tests using the SPSS 27 (IBM Corp., Armonk, NY, USA).

## Results

**Fig. 1:**
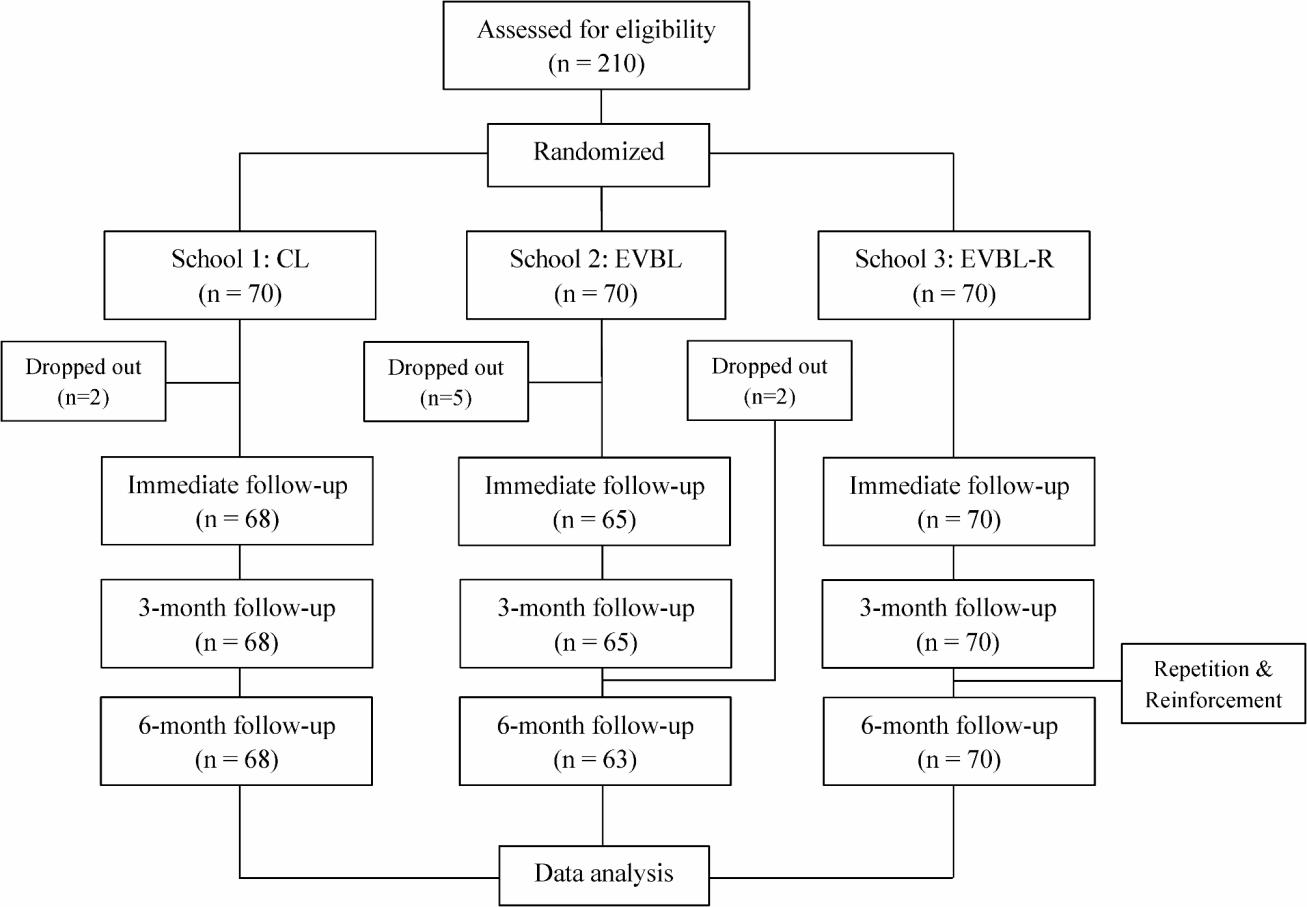
Flow of the participants in the trial. CL: classroom learning, EVBL: edutainment in video-based learning, EVBL-R: edutainment in video-based learning with repetition

A total of 210 students (age range 9.2–10.8 years) were included in this study. The total dropout rate was 4.29%, as shown in Fig. 1. Thus, 201 children remained in the study throughout 6-month follow-up period. The intra- and inter-examiner reliability in OH status evaluation of two examiners was in substaintial agreement. The demographic data and the baseline data are shown in Table 1. The overall mean percentage of the baseline OH knowledge and behavior score was in the range of 31.81–63.9% and 41.83–66.94% respectively, and there was no significant difference among groups.

**Table 1:** Characteristics of demographic data among groups

Characteristics	n(%)			*p-value*
	CL *N* = 68	EVBL *N* = 63	EVBL-R *N* = 70	
**Age**	**Mean ± SD**			0.511
	10.07 ± 0.68	9.89 ± 0.67	10.01 ± 0.73	
**Gender**				0.096
Girl	28(41.2%)	23(36.5%)	38(54.3%)	
Boy	40(58.8%)	40(63.5%)	32(45.7%)	
**Grade**				0.144
4	41(60.3%)	27(42.9%)	35(50%)	
5	27(39.7%)	36(57.1%)	35(50%)	

*Statistically significant difference at *p*-value < 0.05

Comparing the differences in knowledge and behavior scores in receiving OHE through two methods: EVBL vs. CL, it was found that there was a significant higher in two out of three domains of knowledge in the CL, as shown in Table 2. Meanwhile, a significant difference within each group was detected among some different follow-up periods, especially between baseline and immediate follow-up in all domains (*p* < 0.001). The range of 4.12–29.77% and 26.58–53.35% improvement within EVBLs and CL were detected, respectively. For behavior scores, there was no difference among the two methods with The range of 1.19–28.13% and 1.90-15.16% improvement within EVBL and CL were detected, respectively. A significant improvement was detected only within EVBL and EVBL-R groups. The behavioral intention scores were at a moderate to high level (49.8–85%), without a significant difference among groups. The repetition at the 3-month follow-up did not affect the outcomes in EVBL-R group.

**Table 2:** Oral health knowledge, behavior, and intention scores during follow ups

Group Topics	CL (*n* = 68)	EVBL (*n* = 63)	EVBL-*R* (*n* = 70)	*p*-value	CL (*n* = 68)	EVBL (*n* = 63)	EVBL-*R* (*n* = 70)	*p*-value	CL (*n* = 68)		EVBL (*n* = 63)	EVBL-*R* (*n* = 70)	*p*-value	
Knowledge (Mean ± SD)					Behavior (Mean ± SD)					Intention (Mean ± SD)				
**Domain I: General knowledge about dental and gingival diseases including necessity of regular dental check-up visits**														
**(Full score of 8)**					**(Full score of 6)**				**(Full score of 5)**					
Baseline	3.84 ± 1.72				4.09 ± 1.16				4.13 ± 0.12					
	3.97 ± 1.79^**a**^	3.90 ± 1.78^**a**^	3.64 ± 1.59^**a**^	0.498	4.09 ± 1.16	4.11 ± 1.19	4.07 ± 1.15^**a**^	0.979	4.40 ± 0.93^**abc**^		3.97 ± 1.16^**a**^	4.01 ± 1.20	0.056	
Immediate	5.18 ± 1.72^b*^	4.19 ± 2.05^ab*^	4.57 ± 1.81^**b**^	0.010*					4.63 ± 0.60^**b**^		4.44 ± 0.88^**bc**^	4.34 ± 0.96	0.351	
3 months	5.22 ± 1.73^b*^	4.95 ± 2.08^**b**^	4.39 ± 2.21^b*^	0.048	4.21 ± 0.91	4.37 ± 1.15	4.41 ± 1.10^**ab**^	0.282	4.26 ± 0.86^**abc**^		4.14 ± 1.15^**ac**^	4.13 ± 1.14	0.975	
6 months	5.10 ± 1.64^**b**^	3.76 ± 1.77^**b**^	3.79 ± 1.82^**b**^	0.161	4.32 ± 1.01	4.48 ± 1.10	4.54 ± 1.18^**b**^	0.399	4.09 ± 0.96^**ac**^		4.25 ± 1.02^**ac**^	4.20 ± 1.19	0.236	
***p*** **-value**	< 0.001*	< 0.001*	< 0.001*		0.131	0.271	0.006**		< 0.001*		0.046**	0.156		
**Domain II: Oral health care**														
**(Full score of 8)**					**(Full score of 7)**				**(Full score of 5)**					
Baseline	3.51 ± 1.66				3.14 ± 1.36				3.40 ± 0.91					
	3.28 ± 1.44^**a**^	3.33 ± 1.70^**a**^	3.90 ± 1.77^**a**^	0.052	3.10 ± 1.36	2.94 ± 1.24^**a**^	3.36 ± 1.44	0.232	3.31 ± 0.98^**a**^		3.25 ± 1.02^**a**^	3.61 ± 0.69	0.053	
Immediate	5.03 ± 1.61^**b**^	5.38 ± 1.63^**b**^	4.87 ± 1.98^**b**^	0.237					3.81 ± 0.47^**bc**^		3.63 ± 0.77^**bc**^	3.73 ± 0.61	0.480	
3 months	4.50 ± 1.66^**bc**^	4.11 ± 1.79^**cd**^	3.80 ± 2.11^**a**^	0.091	3.57 ± 1.51	3.19 ± 1.41^**ab**^	3.40 ± 1.53	0.216	3.46 ± 0.80^**ac**^		3.30 ± 0.99^**ac**^	3.53 ± 0.76	0.410	
6 months	4.16 ± 1.57^**c**^	3.76 ± 1.77^**ad**^	3.79 ± 1.82^**a**^	0.322	3.57 ± 1.44	3.41 ± 1.56^**b**^	3.61 ± 1.50	0.445	3.31 ± 0.82^**a**^		3.37 ± 0.94^**ac**^	3.59 ± 0.69	0.085	
***p-*** **value**	< 0.001*	< 0.001*	< 0.001*		0.051	0.017**	0.407		< 0.001*		0.003**	0.078		
**Domain III: Diet**														
**(Full score of 5)**					**(Full score of 5)**				**(Full score of 3)**					
Baseline	2.70 ± 1.28				2.56 ± 1.06				2.11 ± 0.98					
	3.16 ± 1.21^a*+^	2.62 ± 1.30^**ac+**^	2.33 ± 1.21^a*^	< 0.001^*^	2.63 ± 1.06	2.60 ± 1.04	2.24 ± 1.07^**a**^	0.406	1.91 ± 1.00^a*^		2.46 ± 0.71^*^	2.00 ± 1.09^**a**^	0.005*	
Immediate	4.00 ± 1.09^b*+^	3.40 ± 1.41^**b+**^	2.90 ± 1.45^b*^	< 0.001^*^					2.32 ± 0.92^**bc**^		2.62 ± 0.61	2.34 ± 0.98^**bc**^	0.231	
3 months	3.15 ± 1.35^a*^	2.73 ± 1.51^**a**^	2.17 ± 1.47^a*^	< 0.001^*^	2.68 ± 1.18	2.79 ± 1.11	2.87 ± 1.91^**b**^	0.614	2.31 ± 0.89^**ac**^		2.35 ± 0.92	2.40 ± 0.95^**bc**^	0.574	
6 months	2.87 ± 1.33^a*+^	2.17 ± 1.42^**c+**^	2.27 ± 1.41^a*^	< 0.001^*^	2.47 ± 1.23	2.71 ± 1.18	2.86 ± 1.22^**b, c**^	0.152	2.19 ± 0.83^**ac**^		2.33 ± 0.90	2.23 ± 1.05^**ac**^	0.390	
***p-value***	< 0.001*	< 0.001*	< 0.001*		0.102	0.119	0.006*		0.002*		0.102	0.009**		

*Statistically significant difference at *p*-value < 0.05. ** Statistically significant difference at *p*-value < 0.05 but no statistically significant difference between group in pairwise comparison

Different alphabets indicate statistically significant difference within group (*p* < 0.05). Same symbol (^*^ or ^+^) indicate statistically significant difference between group (*p* < 0.05)

For OH status, the overall baseline VPI was in the range of poor to fair oral hygiene, and was mostly at a moderate level of inflammation for GI. A significant improvement was detected among groups for VPI (Table 3) or in the range of 32.5-57.08% vs. 36.45–38.79% of CL. at 3- and 6-month follow-up, while there was no significant difference in GI after interventions. Only the EVBL-R group showed a significant improvement in GI between 3 and 6-month follow-ups (*p* < 0.01), as displayed in Table 4.

**Table 3:** Visible plaque index changes during follow ups

Group Topics	CL (*n* = 68)	EVBL (*n* = 63)	EVBL-*R* (*n* = 70)	*p*-value
**Baseline**				
**(Mean ± SD)**	2.11 ± 0.46			
	2.14 ± 0.47^**a**^	2.00 ± 0.42^**a**^	2.19 ± 0.46^**a**^	0.058
Good	0(0%)	0(0%)	0(0%)	
Fair	17(25%)	28(44.4%)	17(24.3%)	
Poor	51(75%)	35(55.6%)	53(75.7%)	
**Immediately post brushing**				
**(Mean ± SD)**	0.99 ± 0.44^**b**^	1.07 ± 0.33^**b**^	1.13 ± 0.54^**b**^	0.159
Good	17(25%)	11(17.46%)	18(25.71%)	
Fair	46(67.65%)	52(82.54%)	45(64.29%)	
Poor	5(7.35%)	0(0%)	7(10%)	
**Recall 3 months**				
**(Mean ± SD)**	1.36 ± 0.38^**c**^	1.35 ± 0.51^**c**^	1.18 ± 0.48^**b**^	0.033**
Good	4(5.88%)	6(9.52%)	14(20%)	
Fair	59(86.76%)	48(76.19%)	52(74.29%)	
Poor	5(7.35%)	9(14.28%)	4(5.71%)	
**Recall 6 months**				
**(Mean ± SD)**	1.31 ± 0.35^c +*^	1.10 ± 0.46^**b+**^	0.94 ± 0.42^c*^	< 0 0.001*
Good	4(5.88%)	15(23.81%)	25(35.71%)	
Fair	63(92.65%)	45(71.43%)	43(61.43%)	
Poor	1(1.47%)	3(4.76%)	2(2.86%)	
**p-value**	< 0.001^*^	< 0.001^*^	< 0.001^*^	

*Statistically significant difference at *p*-value < 0.05. ** Statistically significant difference at *p*-value < 0.05 but no

statistically significant difference between group in pairwise comparison. Different alphabets indicate statistically

significant difference within group. Same symbol (^*^ or ^+^) indicate statistically significant difference between group

**Table 4:** Gingival index changes during follow ups

Group Topics	CL (*n* = 68)	EVBL (*n* = 63)	EVBL-*R* (*n* = 70)	*p*-value
**Baseline**				
**(Mean ± SD)**	1.40 ± 0.29			
	1.28 ± 0.32^a+*^	1.47 ± 0.25^**a+**^	1.44 ± 0.25^a*^	< 0.001*
No inflammation	0(0%)	0(0%)	0(0%)	
Mild inflammation	14(20.59%)	3(4.76%)	6(8.59%)	
Moderate inflammation	54(79.41%)	59(93.65%)	63(90%)	
Severe inflammation	0(0%)	1(1.59%)	1(1.43%)	
**Recall 3 months**				
**(Mean ± SD)**	1.05 ± 0.32^**b**^	1.14 ± 0.31^**b**^	1.13 ± 0.37^**b**^	0.289
No inflammation	0(%)	0(%)	0(%)	
Mild inflammation	34(50%)	32(50.79%)	26(37.14%)	
Moderate inflammation	34(50%)	31(49.21%)	43(61.43%)	
Severe inflammation	0(0%)	0(0%)	1(1.43%)	
**Recall 6 months**				
**(Mean ± SD)**	1.10 ± 0.27^**b**^	1.08 ± 0.21^**b**^	1.04 ± 0.27^**c**^	0.313
No inflammation	0(0%)	0(0%)	0(0%)	
Mild inflammation	31(45.59%)	37(58.73%)	34(48.57%)	
Moderate inflammation	37(54.41%)	26(41.27%)	36(51.43%)	
Severe inflammation	0(0%)	0(0%)	0(0%)	
**p-value**	< 0.001^*^	< 0.001^*^	< 0.001^*^	

*Statistically significant difference at *p*-value < 0.05. Different alphabets indicate statistically significant difference

within group. Same symbol (^*^ or ^+^) indicate statistically significant difference between group

When focusing on the difference among gender and age within each group, some significant different mean scores of knowledge, behavior, behavioral intention, VPI, and GI were detected at only some periods of follow-ups (*p* < 0.001 to 0.042) by the girl and the higher-grade students.

## Discussion

This randomized controlled study evaluated the effectiveness of edutainment use in video-based learning on OHE compared to classroom learning, which is widely used as a traditional method for school-age children. The novelty of EVBL techniques used in this study was that it was age-appropriate content with an up-to-date style that is widely used on trendy platforms in social media. The outcomes were assessed through knowledge, behavior, intention to perform behaviors, and OH status. The results revealed that EVBL was inferior in providing core knowledge but superior in arousing good OH behaviors, as well as improving OH status.

The baseline overall mean percentage of OH knowledge (31.81–63.9%) was similar to the previous studies by Myint et al. [20] (29.6–67.4%), Swe et al. [21] (26.6–71.4%), and Nguyen et al. [22] (33.0-75.6%) which were conducted in the same region, southeast Asia, but when compared to the study in our country it was slightly higher than Potisomporn et al. [23] (23.56–48.69%). The reasons could be the lower grade level of their samples, and the fact that different areas may influence education might be possible in this case. The school-age children in grades 4–5 were chosen in the present study according to their being in the middle of physical, emotional, and cognitive development in the school-age population. They were more reliant on themselves, had the ability to take care of themselves and were also capable of comprehending well in both interventions and the test questions. On the other hand, this could be one of the limitations, as it could not fully represent the entire school-age populations. The mean OH behavior score was higher than those of them; 41.8–66.9% vs.13.2–49.2% [21] and 18.0-66.3% [22]. This may have resulted from different intensities of OH promotions in each country, and it was noticeable that knowledge did not relate to behavior, which was in accordance with Bramantoro et al. [24].

A significant improvement in knowledge in D-I at immediate follow-up and D-III throughout all follow-up periods in the CL group might imply that CL is still the standard method for school-based OHE since it can provide immediate feedback, knowledge exchanges, and more engagement and focus [25]. Besides, the CL in this study was conducted by trained schoolteachers, which proved to be better than the actual OHE class in general government schools [23]. On the contrary, the studies by Hebbal et al. [26] and Mukhi et al. [27] have demonstrated that CL was less effective than audiovisual aids since they could not arouse interest in the subject as much as the video did.

Behavior scores among the two methods of learning were not different. This was in contrast to the previous studies [21, 23, 28] that found significant improvement in behavior scores after receiving OHE when compared to the control group that performed by the obvious inferior methods, including the different content, while our study performed by all the same contents but with the different method, as it was our objective. It is worth noting that improvement was detected only within EVBL and EVBL-R. This might be due to the advantage of the video; the learners could repeat it as often as they want to, being able to pause or replay any part whenever they need to. Furthermore, the video clip was a theory-based intervention; health behavior theories [18, 19] which proved to be a promising technique for OH behavior promotion [19, 29, 30]. It was assumable that the key messages that intended to be communicated with the learner were reached, and it was emphasized more when compared to the CL due to the limited duration of the clip. More importantly, the edutainment features such as music, speech, or lyrics elements and the character of the representation in the clips could have an impact on the learner by developing a positive emotional state, increasing the learner’s retention and focus level, engaging learning physically and mentally, and promoting long term memory recall [31].

According to the theory of Planned Behavior which mentioned that an individual’s behavior was determined by their behavioral intention [32], we intended to include the intention scores in this study. Unfortunately, the result showed the obscured relationship between the two of them. The finding showed that the significantly increased intention scores immediately after the OH session did not affect the behavior score at the following follow-up. It might be inferred that the intention is able to stay for only a short period of time and is not strong enough to change the behavior even in such a short period of time as three months. Moreover, even though the EVBL was more amusing, it could not encourage the extra intentions of the learners since there was no difference from the CL.

It was not surprising that knowledge and behavior scores were immediately improved only after the OHE session and declined by each follow-up, even in the repetition group. The same video clips were repeated and reinforced in the EVBL-R group to evaluate if the repetition enhanced the effectiveness of OHE. However, the fact that repetition only once might not be enough to create the long-term memory that lasts for more than three or six months, as we set out in this study. Previously, it was found that consistent repetition and practice were able to transfer short-term memory into long-term memory [33]. Thus, content repetition alone may not be needed, but repeating the knowledge in practical ways over a period of time consistently, such as by setting up the school system or environment to enhance healthy OH, might further benefit the learners.

The OH status was evaluated by VPI and GI, as it was measurable and reliable and was able to represent the quality of OH practice indirectly. The findings presented the both learning methods in this study could improve the VPI, especially at immediate follow-up, which was similar to previous studies [15, 23, 27, 34]. Nonetheless, it was noted that EVBL seems to have a positive effect on the toothbrushing techniques over CL, as VPI improved over time even in the group without repetition, and the outcome was significant, especially in the last follow-up, and the percentage of good VPI was noticeably 12% higher in the EVBL-R than EVBL group. It was assumable that the improvement of the GI arose from the improvement of VPI consequently. Although the baseline GI were significantly worse in both EVBL groups, it could improve much better than CL group. This was in line with Hebbal et al. [26], Mukhi et al. [27] and Balli et al. [35] whose VBL showed significant improvement in OH status over the CL. In addition, it was possible that the intendable repetition of video clips at three months of follow-up, apart from the replay of clips by the students themselves, might affect the toothbrushing techniques and routines since the GI continued to significantly improve even after a six-month period only in EVBL-R.

It was apparent that all the girls and the higher-grade students showed some significant superior scores when focused on the differences of factors like age and gender within groups at some period of follow-ups in all groups. The previous meta-analysis demonstrated that female students significantly performed better for the examination in all fields of study than the male students, including reading comprehension [36], besides, they had higher achievement motivation [37]. For the grade level, it might be straightforward as the higher grade the students were, the higher their cognitive function, experience, and hand skills tended to be.

The strengths of this cross-sectional study were the integration of educational technology and the OHE, which was timely and relevant to this era, especially after the COVID-19 outbreak, using a three-arm parallel randomized controlled trial with validated measures and structured knowledge test questions. However, the limitation of this study was that the self-administered test questions might cause social desirability bias, especially for behavior and intention parts, and some issues such as diet, regularity for dental visits, self-oral screening, and so forth, limit us from performing a real observation of their behavior and intention. The factors that most influenced the change in a child’s behavior, like parents and different environments among schools, were not included in the study. Even though the contamination within the trial was prevented by separating each group into different schools, external contamination could still occur, particularly in video-based groups, which can cause misinformation from the correct ones. The VBL itself might limited the accessibility in resource-limiting setting and the scalability, as it was still a passive learning method. The more varieties of the video clip styles with a wider age range of the participants may enhance the different result and improve their usage. Finally, the longer period of follow-up possibly magnifies the result regarding the effectiveness and sustainability of the intervention.

## Conclusion

Based on the results of this randomized clinical controlled trial, it can be concluded that the EVBL was comparable to the CL method in encouraging positive OH behaviors, while the CL method was preferable for providing core knowledge. The EVBL was more markedly applicable to how-to content, such as toothbrushing techniques. The repetition of the edutainment video at least every three months might be able to promote a better OH status, and this finding could be applied to any OHE program for school-age children. It is applicable for creating an effective school-based OHE program that integrates both CL and EVBL in one lesson, using CL to provide core knowledge and to evaluate the students understanding as it is able to provide two-way communication, and combine with the EVBL when teaching the how-to technique and expecting the behavior changes. Further study should focus on developing an intervention that is capable of gaining parental involvement, as parents have a powerful impact on the child’s life.

## Electronic supplementary material

Below is the link to the electronic supplementary material.

Supplementary Material 1

Supplementary Material 2

## Data Availability

The datasets used and/or analysed during the current study are available from the corresponding author on reasonable request.

## Abbreviations

CL: Classroom Learning
EVBL: Edutainment in Video-Based Learning
EVBL-R: Edutainment in Video-Based Learning with repetition
GI: Gingival Index
OH: Oral Health
OHE: Oral Health Education
VBL: Video-based learning
VPI: Visible Plaque Index
