# Correction: Effectiveness of edutainment use in videobased learning on oral health education for school-age children: a randomized clinical trial

**DOI:** 10.1186/s12903-026-08980-6

**Published:** 2026-06-30

**Authors:** Wanwisa Lekaram, Pattarawadee Leelataweewud, Pornpailin Kasemkhun

**Affiliations:** Department of Pediatric Dentistry, Faculty of Dentistry, Mahidol University, No.6, Yothi Road, Ratchathewi District, Bangkok, 10400 Thailand

**Correction: BMC Oral Health 25, 381 (2025)**

**https://doi.org/10.1186/s12903-025-05717-9**

In the Ethical Considerations subsection of the Methods section and the Ethical approval and consent to participant section of this article [1], the author inadvertently provided the project number MU-DT/PY-IRB 2022/DT040 instead of the official ethics approval certificate number MU-DT/PY-IRB 2023/034.0606.
