# Supplementary material for: Effectiveness of edutainment use in video-based learning on oral health education for school-age children: a randomized clinical trial
Source: BMC Oral Health. 2025 Mar 13;25:381. doi: 10.1186/s12903-025-05717-9 (PMC11907877; doi:10.1186/s12903-025-05717-9)
Supplement: Supplementary file 2 — Supplementary Material 2 [file 12903_2025_5717_MOESM2_ESM.pdf]

Date________

Participant number________

Oral Health Questionnaire

Part I Demographic data:

1. Age: ________________
2. Gender: ________________
3. Grade: ________________

Part II Questionnaire on oral health knowledge, behavior, and behavioral intention:
(Students were evaluated only 1 domain/session/week)

Domain I: General knowledge about dental and gingival diseases, including the necessity of regular dental check-up visits.

<u>Knowledge</u>

1) Which of the following is <u>not</u> a benefit and importance of teeth?
a. Help enhance personality
b. Help the body receive beneficial nutrients
c. Help with swallowing
d. Help open the bottle cap and tear the snack bag

2) If a permanent molar is decayed to the point of needing extraction, what will be the consequences?
a. It influences pronunciation.
b. The adjacent teeth will tilt and take their place on their own.
c. It has no effect because it's not visible from the outside.
d. It will cause trouble on chewing.
e. It has no impact at all.

3) What is the main cause of tooth decay?
a. Weak enamel
b. There is leftover sugar in the sticky plaque that stuck to the teeth.
c. The toothbrush is not clean.
d. It is a disease that can occur on its own without a specific cause.

4) What are the characteristics of tooth decay?
a. It's a cavity in the tooth.
b. The tooth is a cloudy white.
c. The tooth has brown-orange stains.
d. All above

5) What is plaque?
a. A soft yellowish-white mass that often clings to the tooth near the gum line.
b. A white mass that often accumulates at the tips of the teeth.
c. Brown or black stains that often cling to teeth.
d. I don't know.

6) If you don't brush your teeth properly and plaque accumulates, what will be the consequences?
a. Tooth decay
b. Tartar
c. Gingivitis
d. All above

7) How can tooth decay be prevented?
a. Remove the plaque by brushing your teeth twice a day
b. Use toothpaste that contains fluoride
c. Eat food without added sugar
d. All above

8) When should you visit the dentist?
a. Only when I have symptoms, such as a toothache
b. When all the permanent teeth have come in
c. When seeing cavities in the mouth
d. I should go for regular dental check-ups every 6 months to 1 year.

Behavior

1) I look in the mirror to check my teeth.
a. Yes b. No

2) I always brush my teeth to remove plaque.
a. Yes b. No

3) I went to the dentist when...
a. I found cavity in the tooth.
b. Some teeth are a cloudy white in color.
c. Some teeth have brown-orange stains.
d. When I have a toothache.

4) I checked to see if there was any plaque left behind after brushing my teeth.
a. Yes b. No

5) When did the last time you visit the dentist?
a. In the past 6 months
b. About 1-2 years ago
c. More than 2 years
d. I have never been to see a dentist

6) How many times do you brush your teeth in a day?
a. Not brushing teeth
b. Once
c. 2 times
d. More than 2 times

Behavioral intention

1) I intend to take care of every single tooth as best as I can because I don't want to lose any teeth.
a. Yes b. No

2) I will keep an eye on my teeth.
a. Yes b. No

3) I won't let myself suffer from a toothache.
a. Yes b. No

4) I will brush my teeth clean, without plaque.
a. Yes b. No

5) I intend to inform my parents to take me to the dentist for a dental check-up.
a. Yes b. No

Domain II: Oral health care

Knowledge

1) What a good toothbrush be like?
a. Soft, rounded, flat trim
b. Soft, rounded, rippled trim
c. Hard, pointed, flat trim
d. Hard, rounded ends, rippled trim

2) What is the benefit of having fluoride in toothpaste?
a. For a better toothpaste flavor
b. For effective prevention of tooth decay/ making teeth strong
c. To enhance freshness after brushing your teeth
d. To brush your teeth faster

3) If I had to buy toothpaste myself, what kind of toothpaste would I choose?
a. Choose mint-flavored toothpaste to enhance freshness.
b. Choose herbal toothpaste
c. Choose toothpaste that contains fluoride 1000-1500 ppm
d. Choose toothpaste that contains fluoride 500-1000 ppm

4) Which statement is correct regarding tooth brushing?
a. Brush back and forth in a long line
b. Move back and forth briefly 10 times
c. Brush your teeth alternating between the top and bottom
d. After brushing my teeth, I rinsed my mouth several times until it was clean.

5) To brush your teeth most effectively, how should the bristles be positioned?
a. Place it at an angle of 45 degrees towards the chewing surface of the tooth
b. Place it on the gums more than on the teeth, positioning it perpendicular to the teeth.
c. Place all the toothbrush bristles on the tooth, at a 45-degree angle to the tooth surface.
d. Place the toothbrush at the gum line, with the bristles perpendicular to the teeth.

6) Which of the following is not a principle of the 2-2-2 tooth brushing method?
a. Brush your teeth at least twice a day
b. Brush your teeth for at least 2 minutes each time
c. Rinse with water after brushing your teeth twice

d. Avoid eating for 2 hours after brushing your teeth

7) Why should you avoid eating after brushing their teeth for 2 hours?
a. To keep your mouth fresh for a long time
b. To reduce acid formation and ensure fluoride lasts longer
c. To reduce bleeding from the gums
d. To make teeth whiter

8) When should you brush your teeth?
a. Before breakfast, before dinner
b. After breakfast, before bed
c. After breakfast, before dinner
d. Before breakfast, before bed

Behavior
1) The bristles of your toothbrush being used
a. Soft, rounded, rippled trim
b. Soft, rounded, flat trim
c. Hard, pointed, flat trim
d. Hard, rounded ends, rippled trim

2) The frequency of changing your toothbrushes
a. Every month
b. Every 3 months
c. Every 6 months
d. Once a year
e. Change when the toothbrush bristles are frayed or come off.

3) I use toothpaste that contains 1000-1500 ppm fluoride
a. Yes
b. No
c. I don't know/I'm not sure.

4) The amount of toothpaste you used for one brushing
A. Full length of the brush
b. Half the length of the brush
c. As small as a pea
d. Light touch

5) The posture for brushing yourteeth
a. Rub back and forth horizontally
b. Swipe up and down
c. Wipe back and forth horizontally while alternating up and down
d. Rub in a circular motion
e. Do all the things mentioned above

6) Currently, which areas do you brush the teeth? (You can choose more than one option)
a. On the cheek side
b. On the side of the tongue or the palate
c. Chewing side
d. All the aspects mentioned

7) After brushing my teeth...
a. I haven't eaten anything for 2 hours
b. I eat immediately
c. I have breakfast
d. I fell asleep immediately

Behavioral intention

1) I will choose the toothbrush to use by myself.
a. Yes b. No

2) I will choose toothpaste that contains fluoride at 1,000-1,5000 ppm.
a. Yes b. No

3) I intend to brush my teeth properly to keep them clean.
a. Yes b. No

4) I will brush my teeth according to the 2-2-2 principle, covering all aspects.
a. Yes b. No

Domain III: Diet

Knowledge

1) Which type of food does not contain added sugar?
a. Crispy snacks
b. Chocolate milk
c. Salted roasted peanuts.
d. Vanilla ice cream.

2) What should I eat as a snack to prevent tooth decay?
a. Fruit-flavored yogurt
b. Freshly squeezed juice
c. Fruit-flavored jelly
d. Fresh fruits

3) Which statement is correct regarding snacking?
a. Snack on foods that do not contain added sugar, such as fresh fruits and dumplings.
b. You can eat jelly as often as you want if you brush your teeth every night before bed.
c. Drinking flavored milk or fruit juice as a snack instead of fresh fruit.
d. You shouldn't have snack; if you're hungry, you should hold out until the main meal.

4) Why is it important to read the nutrition label before buying snacks?
a. To check the cost-effectiveness of the nutrients received against the price
b. To know the main ingredients of the dessert if it made from plants or animal products.
c. Some types of sweets contain added sugar, which is a cause of tooth decay.
d. to study the methods for storing leftover snacks.

5) If the key ingredients of a certain type of crispy snack are as follows: "Potatoes 52%, rice bran oil 32%, wheat flour 7%, seasoning 5% and flavoring agents 4%" Does this snack cause tooth decay?
a. Not possible, as there is no sugar added.
b. Not really, because the main ingredient is potatoes, and eating it won't cause tooth decay.

c. Yes, because all kinds of sweets can cause tooth decay.
d. Sure, since this type of snack is crispy potato chips, it tends to get stuck in your teeth and can decay.

Behavior

1) I eat sweets/desserts between meals every day.
a. 1 time
b. 2 times
c. 3 times
d. More than 3 times.

2) The time when I eat dessert...
a. Mid-morning snack
b. Afternoon snack
c. Snack before having dinner
d. Eating immediately after a big meal

3) The snack I chose to eat...
a. Fresh fruit
b. Fruit juice
c. Snack packets, crispy snacks
d. Thai desserts
e. Fried foods, such as French fries and fried chicken
f. Soft drinks, yogurt drinks, malt extract beverages
g. Dumplings, steamed buns, sausages, meatballs

4) I read the nutrition label before choosing to buy food/snacks.
a. Yes
b. No

5) I think about calculating the amount of sugar I consume each day.
a. Yes
b. No

Behavioral intention

1) I will eat healthy snacks without added sugar and not sticky to the teeth.
a. Yes b. No

2) I will read the nutrition label every time before choosing to buy food.
a. Yes b. No

3) I will not consume more than 6 teaspoons of sugar a day.
a. Yes b. No
